# Age and traumatic brain injury as prognostic factors for late-phase mortality in patients defined as polytrauma according to the New Berlin Definition: experiences from a level I trauma center

**DOI:** 10.1007/s00402-020-03626-w

**Published:** 2020-10-17

**Authors:** V. Weihs, V. Heel, M. Dedeyan, N. W. Lang, S. Frenzel, S. Hajdu, T. Heinz

**Affiliations:** Department of Orthopedics and Trauma Surgery, Division of Trauma Surgery, Medical University of Vienna, Währinger Gürtel 18-20, 1090 Vienna, Austria

**Keywords:** Polytrauma, Outcome, Traumatic brain injury, Mortality

## Abstract

**Background:**

The rationale of this study was to identify independent prognostic factors influencing the late-phase survival of polytraumatized patients defined according to the New Berlin Definition.

**Methods:**

Retrospective data analysis on 173 consecutively polytraumatized patients treated at a level I trauma center between January 2012 and December 2015. Patients were classified into two groups: severely injured patients (ISS > 16) and polytraumatized patients (patients who met the diagnostic criteria for the New Berlin Definition).

**Results:**

Polytraumatized patients showed significantly lower late-phase and overall survival rates. The presence of traumatic brain injury (TBI) and age > 55 years had a significant influence on the late-phase survival in polytraumatized patients but not in severely injured patients. Despite the percentage of severe TBI being nearly identical in both groups, severe TBI was identified as main cause of death in polytraumatized patients. Furthermore, severe TBI remains the main cause of death in polytraumatized patients > 55 years of age, whereas younger polytraumatized patients (< 55 years of age) tend to die more often due to the acute trauma.

**Conclusion:**

Our results suggest that age beyond 55 years and concomitant (severe) TBI remain as most important influencing risk factor for the late-phase survival of polytraumatized patients but not in severely injured patients.

**Level of evidence:**

Prognostic study, level III.

## Introduction

Recent literature has detected prognostic factors regarding the survival of trauma patients and the outcome of polytraumatized patients. The main focus of this publications was on specific types of trauma [1–6] or specific types of injuries [7, 8]. The term “Polytrauma” has been frequently defined as patients with a high injury severity score (ISS) without regard to their pathophysiological conditions. An injury severity score (ISS) greater than 16 points has been described to result in a mortality risk above 10% [9] which determines an internationally accepted threshold for “polytrauma”. Without a clear definition of polytrauma it remains challenging to compare clinical characteristics, outcomes and potential prognostic factors on the survival of polytrauma patients [10]. When defining polytrauma both the anatomical as well as the physiological parameters should be included [11]. A well-known definition of polytrauma is the New Berlin Definition that adds at least one of five standardized physiological conditions to the definition of ISS ≥ 16 and at least two different affected body regions with an AIS ≥ 3 [12]. The New Berlin Definition has been shown to be feasible and applicable for polytrauma patients [12–15]. Rau et al. [13] showed that polytrauma patients identified according to the New Berlin Definition had significantly higher ISS scores and significantly higher mortality rates.

To our knowledge there is little data on possible prognostic factors influencing especially the late-phase survival of polytraumatized patients classified according to the New Berlin Definition when compared to severely injured patients (ISS > 16 points).

Our study addresses this issue and compares patients who were identified as polytraumatized patients according to the criteria of the New Berlin Definition or defined either as severely injured patients with an ISS > 16 points without regard to their pathophysiological conditions:

- Do patients defined as polytraumatized according to the New Berlin Definition show different clinical characteristics, trauma mechanisms or different injury characteristics?
- Are there any potential prognostic factors especially for the late-phase survival of these polytraumatized patients?

## Methods

### Inclusion criteria

In this study 337 consecutive patients who were admitted to our hospital with critical injuries were enrolled retrospectively from January 2012 to December 2015. Patients with an ISS > 16 points and an AIS > 3 and at least 2 or more different body regions affected were included. Patients with isolated traumatic brain injuries (*n* = 101), patients with minor injuries (AIS < 3 or ISS < 17) (*n* = 45) and patients younger than 18 years of age (*n* = 18) were excluded, leading to 173 remaining patients.

The included 173 patients were divided into two groups:

- severely injured patients with an ISS > 16 points as well as an AIS > 3 in one body region and at least 2 or more different body regions affected (*n* = 80) and
- polytraumatized patients that were classified according to the New Berlin Definition (*n* = 93): AIS > 3 points for two or more different body regions with the addition of at least one of five standardized physiological conditions (hypotension (SBP < 90 mmHg), unconsciousness (GCS score < 8), acidosis (BE < − 6.0), coagulopathy (aPTT > 40 s or INR > 1.4) or age (> 70 years).

Three time-dependent events for the analysis of mortality were defined: acute-phase death (death within the first 24 h or on arrival at the hospital), late-phase death (death after the first 24 h within the hospital stay) and overall-death [death of the disease (DOD)-defined as death at any time within the hospital stay]. Data analysis focused on potential prognostic factors regarding the late-phase survival. Possible prognostic factors such as severe TBI, age, injury severity and trauma mechanism were detected.

### Statistical analysis

Data analysis focused on potential prognostic factors regarding the late-phase survival in severely injured patients (ISS > 16 points) compared to polytraumatized patients (determined according to the Berlin Definition). Continuous variables are presented as means and standard deviations or medians and interquartile ranges. Categorical variables are provided with percentages. Descriptive statistics were used for demographic variables and clinical characteristics. Trauma mechanisms, injury characteristics and severity of injuries (classified with the AIS score) were examined. For detection of associations between qualitative variables a chi-square test was performed. For comparison between categorical and continuous variables the Student *t* test was done. A two-sided *p* value of less than 0.05 was considered to indicate statistical significance. The Kaplan–Meier method was used to provide survival estimates, which were assessed with a log-rank test. For analysis of prognostic factors on the late-phase survival, patients who died of unrelated causes or within the acute-phase were considered to have been censored. All statistical analysis was performed using IBM SPSS Statistics Version 26.0.

## Results

### Study population

One-hundred-and-seventy-three severely injured patients were enrolled consecutively from January 2012 to December 2015: 126 patients (72.8%) were male, 47 patients (27.2%) female with an average age at time of trauma of 45.12 years (range from 18 to 93 years). The baseline characteristics such as gender, age and clinical symptoms, the trauma mechanism as well as the short-term outcomes were reported (Table 1). No gender differences between the two groups were detected. The mean age in both groups was nearly identical: 46.54 years of age (± 20.34) in polytraumatized patients and 43.48 years of age (± 16.72) in severely injured patients (*p* = 0.286). Despite this, the percentage of geriatric patients (> 65 years of age) was significantly higher in polytraumatized patients (21.5% vs. 10.0%; *p* = 0.041).

**Table 1:** Clinical characteristics of severely injured patients (*n* = 80; 46.2%) compared to polytraumatized patients (*n* = 93; 53.8%)

	Polytraumatized Patients (*n* = 93)	Severely Injured Patients (*n = *80)	*p* value
Gender			
Women	21 (22.6%)	26 (32.5%)	0.144
Men	72 (77.4%)	54 (67.5%)	
ISS	35 (18–75)	28 (17–75)	< 0.001*
Age	47 (18–93)	43 (18–90)	0.286
Trauma mechanism			
Motor vehicle accident	60 (64.5%)	56 (70%)	0.693
Fall from greater height	22 (23.7%)	16 (20%)	
Fall from lesser height	6 (6.5%)	2 (2.5%)	
Penetrating injury	4 (4.3%)	5 (6.3%)	
Other mechanism	1 (1.1%)	1 (1.3%)	
Severe TBI (AIS ≥ 3)	56 (60.2%)	38 (47.5%)	0.094
Thoracic injury	88 (94.6%)	64 (80%)	0.003*
Abdominal injury	45 (48.4%)	32 (40%)	0.268
Pelvic injury	36 (38.7%)	14 (17.5%)	0.002*
Acute-phase mortality	17 (18.3%)	5 (6.3%)	0.018*
Late-phase mortality	18 (19.4%)	3 (3.8%)	0.002*
Overall mortality	35 (37.6%)	8 (10%)	< 0.001*

*ISS* injury severity score, *TBI* traumatic brain injury

* Chi-square test and independent *t* test. A *p* value less than 0.05 was considered to indicate statistical significance

### Injury characteristics and injury severity

The main mechanism of injury was a motor vehicle accident followed by a fall from greater height (> 3 m) in both groups even though not statistically significant. In polytraumatized patients a suicidal attempt was documented more often (18.3% vs. 8.8%; *p* = 0.071). The presence of traumatic brain injury (TBI) was similar in both groups (66.7% vs. 61.3%; *p* = 0.459), whereas severe TBI (AIS ≥ 3) was seen more often in polytraumatized patients (60.2% vs. 47.5%; *p* = 0.094) without any statistical difference. Thoracic injuries and pelvic injuries were seen significantly more often in polytraumatized patients. No differences in the presence of abdominal injuries, injuries of the spine or injuries of extremities could be detected in both groups (Table 1).

**Fig. 1:**
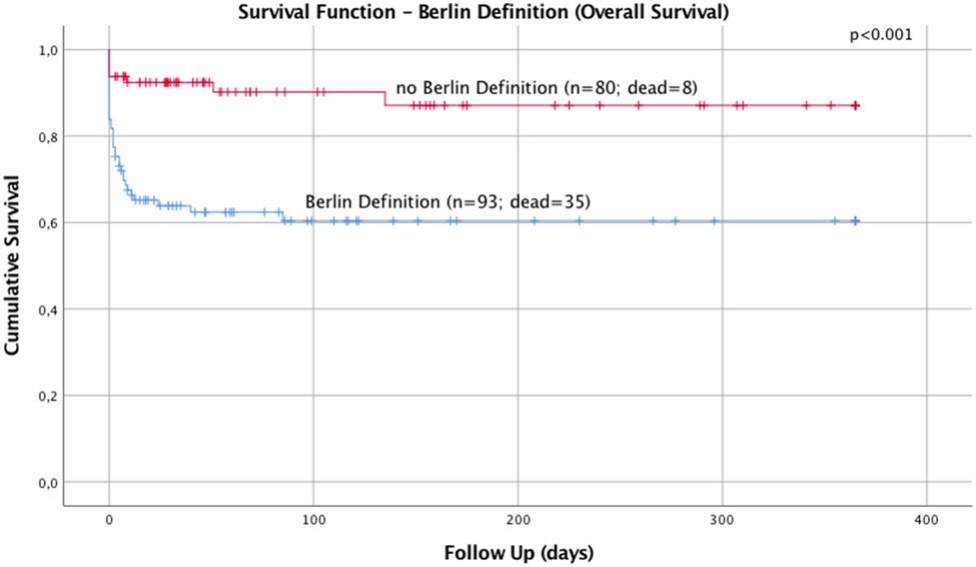
Overall survival rates in polytraumatized patients compared to severely injured patients

Polytraumatized patients showed significantly higher ISS scores (35.29 vs. 27.54; *p* < 0.001). In these patients a resuscitation had to be performed more often (*p* = 0.013) and they showed significantly lower survival rates in the acute phase (*p* = 0.018) and the late phase (*p* = 0.002). Polytraumatized patients showed an overall mortality rate of 37.6% compared to severely injured patients who showed an overall mortality rate of 10% (*p* < 0.001; Fig. 1).

### Late-phase survival

No influence of gender, suicidal attempt, thoracic, abdominal or pelvic injuries on the late-phase survival could be detected in both groups. The presence of TBI had a significant influence on the late-phase survival in polytraumatized patients (*p* = 0.014) but not in severely injured patients (*p* = 0.109). Regarding the presence of severe TBI a significant influence on the late-phase survival could be detected in both groups. The AUC for predicting late-phase mortality using the age on admission was 0.717 (*p* < 0.001). Based on these findings our results suggest an optimal age cut-off value of 55 years on admission to predict late-phase mortality. Kaplan–Meier curves were created for late-phase mortality based on age on admission. Results of the log-rank test (*p* = 0.001) showed that age > 55 years was a predictor of late-phase survival in polytraumatized patients but not in severely injured patients (*p* = 0.001).

Despite the percentage of severe TBI being nearly identical in both groups, severe TBI was identified as main cause of death in polytraumatized patients. Furthermore, severe TBI remains the main cause of death in polytraumatized patients > 55 years of age, whereas younger polytraumatized patients (< 55 years of age) tend to die more often due to the acute trauma (*p* = 0.002).

### Limitation

This retrospective non-randomized, single-center analysis has the characteristic limitations of registry data and post hoc analyses. There are no data on functional outcome and quality of life parameters available. Furthermore, long-term mortality was not evaluated in this study. Due to the retrospective design of this study there might be an inherent selection bias. The strength of this study and sign of quality is the careful analysis of data in all consecutively included patients.

## Discussion

Our study contributes new insights regarding prognostic factors in patients defined as “polytrauma” according to the New Berlin Definition. Although a decline in trauma related deaths can be seen over time, trauma remains one of the leading causes of death world-wide [16].

First, we found an increase in the mean age of our polytraumatized patients compared to previous studies [17], reflecting the rapidly aging population world-wide. As already well documented, mortality rates in trauma patients increase with age, even more in the sixth and seventh decade of life [18, 19]. Especially, elderly patients, mainly > 75 years of age show a peak in mortality which is seen around day 6 after the initial trauma [19]. The New Berlin Definition uses an age > 70 years as physiological condition for polytraumatized patients with an increased mortality rate [12–15]. This cut-off of > 70 years was chosen due to a higher mortality rate in this age group [12]. In our study population even younger polytraumatized patients with an age of > 55 showed significantly lower late-phase survival rates when compared to severely injured patients.

Secondly, the presence of TBI had a significant influence on the late-phase survival in polytraumatized patients but not in severely injured patients (although the incidence of TBI was nearly identical in both groups of our patients). These results suggest that concomitant TBI in polytraumatized patients leads to higher mortality rate reflecting once again the vulnerability of these patients. An increase in the incidence of TBI in elderly trauma patients mainly caused by road injuries and falls has been shown [20]. Recent studies suggest TBI as a strong predicting factor for the survival in trauma patients [21, 22]. Even more, concomitant injuries seem to have a significant effect on the mortality in patients with moderate TBI [23]. Severe TBI had a significant influence on the late-phase survival in both groups of our patients and was detected more often in polytraumatized patients. In accordance with the literature [4, 5, 24–28] severe TBI remains the most common cause of death in 61.9% of all late-phase deaths in our patients. Furthermore severe TBI iwas the main cause of death in polytraumatized patients > 55 years of age, whereas younger polytraumatized patients (< 55 years of age) tend to die more often due to the acute trauma.

Thirdly, our study confirms that the New Berlin Definition is feasible and applicable for polytrauma patients. When comparing mortality rates of polytraumatized and severely injured patients higher mortality rates in polytraumatized patients (37.6% vs. 10%) were observed in our study population.

Our results suggest a strong influence of severe TBI and an age > 55 years on the late-phase survival of polytraumatized patients meeting the New Berlin Definition.

## Conclusion

Our results suggest that even younger polytraumatized patients (> 55 years of age) as well as polytraumatized patients with concomitant TBI have a higher late-phase mortality compared to severely injured patients.

## Data Availability

The data used/analyzed is available from the corresponding author upon reasonable request.
